# Human Umbilical Mesenchymal Stromal Cells Mixed with Hyaluronan Transplantation Decreased Cartilage Destruction in a Rabbit Osteoarthritis Model

**DOI:** 10.1155/2021/2989054

**Published:** 2021-10-21

**Authors:** Yu-Hsun Chang, Dah-Ching Ding, Kun-Chi Wu

**Affiliations:** ^1^Department of Pediatrics, Hualien Tzu Chi Hospital, Buddhist Tzu Chi Medical Foundation and Tzu Chi University, Hualien, Taiwan; ^2^Department of Obstetrics and Gynecology, Hualien Tzu Chi Hospital, Buddhist Tzu Chi Medical Foundation and Tzu Chi University, Hualien, Taiwan; ^3^Department of Obstetrics and Gynecology, College of Medicine, Tzu Chi University, Hualien, Taiwan; ^4^Department of Orthopedics, Hualien Tzu Chi Hospital, Buddhist Tzu Chi Medical Foundation and Tzu Chi University, Hualien, Taiwan

## Abstract

Osteoarthritis (OA), the most common type of arthritis, causes pain in joints and disability. Due to the absence of ideal effective medication, stem cell transplantation emerges as a new hope for OA therapy. This study is aimed at evaluating the capability of human umbilical cord mesenchymal stromal cells (HUCMSCs) mixed with hyaluronan (HA) to treat osteoarthritis in a rabbit model. Differentiation capability of HUCMSCs, magnetic resonance image examination, and immunohistochemistry of the cartilage after transplantation of HUCMSCs mixed with HA in a rabbit OA model were explored. HUCMSCs exhibited typical mesenchymal stromal cell (MSC) characteristics, including spindle-shaped morphology, surface marker expressions (positive for human leukocyte antigen- (HLA-) ABC, CD44, CD73, CD90, and CD105; negative for HLA-DR, CD34, and CD45), and trilineage differentiation (chondrogenesis, adipogenesis, and osteogenesis). The gene expression of SOX9, type II collagen, and aggrecan in the HUCMSC-derived chondrocytes mixed with HA was increased after in vitro chondrogenesis compared with HUCMSCs. A gross and histological significant improvement in hyaline cartilage destruction after HUCMSCs mixed with HA was noted in the animal model compared to the OA knees. The International Cartilage Repair Society histological score and Safranin O staining were significantly higher for the treated knees than the control knees (*p* < 0.05). Moreover, the expression of MMP13 was significantly decreased in the treated knees than in the OA knees. In conclusion, HUCMSCs mixed with HA in vitro and in vivo might attenuate the cartilage destruction in osteoarthritis. Our study provided evidence for future clinical trials.

## 1. Introduction

Osteoarthritis (OA) is the most common type of arthritis. It causes disability and pain in joints [1]. The increasing aging population and increased obesity prevalence equally increased the prevalence of OA worldwide [2]. Thus, OA increases both personal and socioeconomic burden and has become a public health challenge [3]. Current nonsurgical treatment of OA includes nonpharmacological and pharmacological methods. However, the current pharmacological treatment only focuses on symptom relief and has no effects on regeneration or reconstruction [4].

Stem cell therapy is an emerging therapy. Stem cells have self-renewal capacity, multilineage differentiation capability, and immunomodulatory properties [5]. Among stem cells, autologous mesenchymal stromal cells (MSCs) derived from bone marrow and adipose tissue are the most investigated agents in OA [6]. The mechanism of stem cell therapy for OA is still elusive. Clinical trials showed that stem cell therapy could relieve joint pain, although limited evidence of cartilage thickness change was noted [7].

Human umbilical cord MSCs (HUCMSCs) are obtained during childbirth and are usually regarded as medical waste. The procedure of collection is less invasive and not painful when compared to bone marrow and adipose tissue collection [8]. The chondrogenic potential of HUCMSCs has been studied; however, in vivo studies have shown inconsistent results [9–11]. Therefore, we need to investigate HUCMSCs' capability for repairing cartilage.

Hyaluronic acid (HA) is one of the glycosaminoglycans and is naturally born in cartilage tissue [12]. HA composes a microenvironment for cartilage homeostasis and differentiation. Previously, we and other groups used 4% HA and HUCMSCs in a pig OA model and showed that it could downregulate MMP13 and upregulate aggrecan, type II collagen, and SOX9 [10, 13]. In addition, we previously reported that 25%, 50%, and 75% HA mixed with infrapatellar fat pad MSC could have chondrogenic potentials [14]. The percentage of HA that would be useful in stem cell transplantation needs to be further elucidated.

This study is aimed at evaluating the capability of HUCMSCs and HA to treat osteoarthritis in a rabbit model. Differentiation capability of HUCMSCs, magnetic resonance image of cartilage, and immunohistochemistry of the cartilage after transplantation of HUCMSCs mixed with HA were explored.

## 2. Materials and Methods

### 2.1. Isolation and Expansion of Human Umbilical Cord Mesenchymal Stromal Cells (HUCMSCs)

The protocols for this study were approved by the Research Ethics Committee of Hualien Tzu Chi Hospital (IRB 100-166). Informed written consent was obtained from all patients before their enrollment in this study.

The detailed experimental protocol of HUCMSCs had been reported previously with slight modifications [6, 8, 15]. Briefly, the human umbilical cord (10 cm in length) was collected in sterile boxes containing normal saline (SinDong Co., Taoyuan, Taiwan) and sent to the laboratory within 24 h. The Ca^2+^- and Mg^2+^-free phosphate-buffered saline (PBS, Biowest, Nuaille, France) was used to wash the human umbilical cord three times. Scissors were used to cut the cord in a midline direction. The umbilical vein, artery, and surrounding membrane were dissociated with the WJ. The jelly was cut into small pieces and treated with type I collagenase (Sigma, St Louis, MO, USA). The jelly pieces were incubated for 14–18 h in the incubator set with 37°C and 95%-air/5% CO_2_ humidified atmosphere. The explants were cultured in low-glucose Dulbecco's Modified Eagle's Medium (DMEM-LG) (Sigma) containing 10% fetal bovine serum (FBS, Biological Industries, Kibbutz Beit Haemek, Israel) and penicillin/streptomycin. The explants were left undisturbed for 5–7 days to allow cells to migrate from the explants. The resulting HUCMSCs were appointed as passage 1. The medium was changed twice a week. The cells were passaged when 90% of confluence was reached.

### 2.2. Flow Cytometry

The surface markers of HUCMSCs of passages 3 and 4 were checked by flow cytometry. The cells were detached by PBS and Accutase (Millipore, Billerica, MA, USA). Then, the cells were washed with PBS containing 0.1% sodium azide (Sigma) and 2% bovine serum albumin (Sigma). The respective antibodies conjugated with phycoerythrin or fluorescein isothiocyanate were used, including HLA-ABC, HLA-DR, CD29, CD34, CD44, CD45, CD90, and CD105 (BD Pharmingen, Franklin Lakes, NJ, USA). After incubation for 1 hour, the cells were analyzed by a flow cytometer (Becton Dickinson, San Jose, CA, USA).

### 2.3. Adipogenesis Induction

The adipogenic medium consisted of DMEM supplemented with 10% FBS, 0.5 mmol/L isobutylmethylxanthine, 5 *μ*g/mL insulin, 60 *μ*mol/L indomethacin, and 1 *μ*mol/L dexamethasone (Sigma). A total of 5 × 10^4^ HUCMSCs were seeded onto a 12-well plate with adipogenic medium and cultured for 14 days. The medium was changed every 3 days. After 14 days of adipogenic differentiation, Oil red O (Sigma) was used to stain intracytoplasmic oil droplets that were indicative of adipocytes.

### 2.4. Osteogenesis Induction

The osteogenic medium consisted of DMEM supplemented with 10 mmol/L *β*-glycerol phosphate, 10% FBS, 0.1 *μ*mol/L dexamethasone, and 50 *μ*mol/L ascorbic acid. A total of 1 × 10^4^ HUCMSCs were seeded into each well of a 12-well plate with the above medium. The medium was changed every 3 days. Following osteogenic differentiation for 14 days, Alizarin red (Sigma) was used to stain intracytoplasmic mineral depositions that were indicative of osteoblasts.

### 2.5. Chondrogenesis Induction

The chondrogenic medium consisted of DMEM, 10% FBS, 50 *μ*g/mL ascorbic acid-2-phosphate (Sigma-Aldrich), 10 ng/mL transforming growth factor-*β*1 (PeproTech Inc., Rocky Hill, NJ), and 6.25 *μ*g/mL insulin (Sigma-Aldrich). The 25 × 10^6^ cells of HUCMSCs were seeded with a total volume of 0.75 *μ*L chondrogenic medium plus 30 *μ*L of the basic medium onto the bottom of dry 15 mL test tubes (BD Pharmingen). The tube was placed in a 37°C and humidified CO_2_ incubator. The media were changed every 2 days. After 3 weeks of culturing, micromass cartilages were formed. The micromass cartilages were fixed in 4% paraformaldehyde at 4°C for one day. In addition, PBS was used to wash the cartilage, transfer it to 70% ethanol (see Histology).

### 2.6. Real-Time Reverse-Transcriptase Polymerase Chain Reaction

Total ribonucleic acid (RNA) from the undifferentiated HUCMSCs, HUCMSCs with trilineage differentiations, and chondrogenesis of HUCMSCs with or without HA was extracted from the cultures using a RNeasy Protect Mini Kit (Qiagen, Hilden, Germany). A SuperScript III One-Step RT-PCR kit (Invitrogen, Grand Island, NY, USA) was used for reverse-transcriptase polymerase chain reaction (RT-PCR) (Roche Applied Science, Penzberg, Bavaria, Germany). Quantification of gene expression was analyzed in a quantitative real-time PCR detection system (ABI StepOnePlus System; Applied Biosystems, Foster City, CA, USA). The primers (Invitrogen) were *PPARγ* (adipogenic marker) (forward, 5′-AGCCTCATGAAGAGCCTTCCA-3′; reverse, 5′-TCCGGAAGAAACCCTTGCA-3′), *OPN* (osteogenic marker) (forward, 5′-AGGAGGAGGCAGAGCACA-3′; reverse, 5′-CTGGTATGGCACAGGTGATG-3′), *SOX9* (chondrogenic marker) (forward, 5′-ACACACAGCTCACTCGACCTTG-3′; reverse, 5′-GGGAAT TCTGGTTGGTCCTCT-3′), aggrecan (chondrogenic marker) (forward, 5′-GAGATGGAG GGTGAGGTC-3′; reverse 5′-ACGCTGCCTCGGGCTTC-3′), type II collagen (*COL2A1*; chondrogenic marker) (forward, 5′-GGACTTTTCTCCCCTCTCT-3′; reverse, 5′-GACCCGAAGGTCTTACAGGA-3′), *MMP13* (catabolic marker) (forward, 5′-CTT GAT GCC ATT ACC AGT C-3′; reverse, 5′-GGT TGG GAA GTT CTG GCC A-3′), and glyceraldehyde-3-phosphate dehydrogenase (*GAPDH*; internal control and housekeeping gene) (forward, 5′-GAAGGTGAAGGTCGGAGTC-3′; reverse, 5′-GAAGA TGGTGATGGGATTTC-3′). QPCR software (Applied Biosystems) was used to measure the threshold cycle (Ct) value. The 2^−*ΔΔ*Ct^ method was used to normalize the Ct values to GAPDH [16].

### 2.7. A Rabbit OA Model

All experimental protocols were approved by the Institutional Animal Care and Use Committee of Hualien Tzu Chi Hospital (107-51). All methods were carried out in accordance with relevant guidelines and regulations. The study was carried out in compliance with the ARRIVE guidelines.

Totally, six New Zealand White (NZW) rabbits (14 months old) were used in this study. The anterior cruciate ligament transection (ACLT) procedure was performed for bilateral knees of the hind legs of each rabbit after general anesthesia. The right knee was for HUCMSC treatment, and the other one was for control (injected with 500 *μ*L normal saline). Eight weeks after the operation, all the rabbits received 1 × 10^7^ HUCMSCs plus 0.05% HA therapy (total volume: 500 *μ*L) for 6 rabbits. Six weeks after HUCMSC therapy, all the rabbits underwent the first MRI study for both knees. Twelve weeks after MSC therapy, all the rabbits underwent the second MRI study for both knees again. After sacrificing, the OA severity was evaluated both using imaging and pathology diagnosis.

### 2.8. Surgical Procedure [16, 17]

An intravenous injection of xylazine (3 mg/kg) and ketamine (25 mg/kg) was used to anesthetize all rabbits. Betadine solution was used to disinfect both knees. An arthrotomy was performed after a medial parapatellar incision. Dislocated laterally the patella and full flexion of the knee were performed. Expose the ACL and use a no. 15 blade to transect. Irrigate the joint with sterile saline and close. Use a running structure of 4-0 Prolene to approximate the capsule and the synovium was performed. The skin was sutured and closed. Pain relief after operation was reached by giving oral meloxicam 0.2 mg/kg/day for 7 days.

### 2.9. Magnetic Resonance Imaging (MRI) Study

Six weeks after HUCMSC therapy, all the rabbits underwent the first MRI (0.3 T, AIRIS 2, Hitachi Medical Systems, Japan) study for both knees. Twelve weeks after MSC therapy, all rabbits underwent the second MRI study for both knees again. During the exam, the rabbits were euthanized. The severity of each joint was evaluated and classified from grade one to six, divided into normal, minimal fibrillation, overt fibrillation, erosion 0-2 mm, erosion 2-5 mm, and erosion > 5 mm^16^. Two observers (KCW and YHC) independently reviewed the pictures and gave grading. The knee was divided into AP and lateral view. Cartilage integrity was reviewed on AP and lateral view, based on the most severe defect on the joint surface.

### 2.10. Tissue Harvest

 

### 2.11. Macroscopic Examination

The knees were removed after the rabbits were euthanized with an intramuscular injection of xylazine (6 mg/kg) and ketamine (50 mg/kg). Expose the surface of the distal femur and proximal tibia and examine grossly. India ink 2 mL was injected on the tibial plateau and waited for 2 min and washed with saline. The staining of the tibial plateau was inspected microscopically.

#### 2.11.1. Histological Evaluation

The proximal tibial plateau and the distal femora were removed. 10% buffered formalin (Sigma) was used to fix for 48 h. 10% EDTA (Gibco, Grand Island, NY, USA) was used to decalcify the specimen for 2 weeks. Then, the specimen was cut into four pieces. All pieces were embedded in paraffin. Serial sagittal sections were stained with Safranin O (Sigma) and hematoxylin and eosin (H&E, Sigma). Histological images were obtained by microscopy. The grading of histology change was assessed by the ICRS histological score [18].

#### 2.11.2. Immunohistochemistry

The articular sections of tibia were rehydrated and blocked with 3% hydrogen peroxide (Sigma). Type II collagen was retrieved with a mixture of 1 mg/mL of Pronase in PBS (pH 7.4; Sigma) and 2.5% hyaluronidase (Sigma) for 1 h at 37°C. Then, the specimen was treated with 1 mg/mL of pepsin (Sigma) in Tris HCl (MD Bio, Taipei, Taiwan) for 15 min at 37°C.

The paraffin sections received serial sections with 5 *μ*m thickness. Then, sections were blocked with Ultra V block (Thermo Scientific, Fremont, CA, USA) for 10 min and incubated with primary antibodies against type II collagen and aggrecan (1 : 200, GeneTex, Irvine CA, USA) at 37°C for 4 h. The secondary antibodies were incubated with biotin-labeled goat anti-rabbit immunoglobulin (Dako, Carpinteria, CA) and horseradish peroxidase-conjugated streptavidin (Biocare Medical) for 30 min. Finally, the sections were stained with a 3,3-diaminobenzidine solution. The hematoxylin (Sigma) was used to counterstain the slides. We used ImageJ software to count the IHC intensity of the cartilage area.

Immunohistochemistry staining of TNF-*α* (1 : 200, Novus Biologicals, Centennial, CO, USA), IL1-*β* (1 : 200, Abbexa, Cambridge, UK), IL-6 (1 : 200, Taiclone Biotech Corp. Taipei, Taiwan), and MMP13 (1 : 200, Novus Biologicals, Centennial, CO, USA) was used to evaluate the inflammatory and catabolic status of the joints in each group. A total of 50 cells were counted at random from three areas and showed an average positive staining cell number [19].

### 2.12. Statistical Analysis

The severity of MRI study between the OA knees and MSC-treated knees was compared using Pearson's chi-squared test to determine whether there was a statistically significant difference. Nonparametric tests (Mann–Whitney *U* test) were used to compare the histopathological grade among the two groups. All data were expressed as median and range. When the *p* value was <0.05, they were considered statistically significant. SPSS version 25 (IBM, New York, NY, USA) was used to perform all statistical analyses.

## 3. Results

### 3.1. HUCMSCs Exhibit MSC Characteristics

#### 3.1.1. Surface Marker Expression

**Figure 1:**
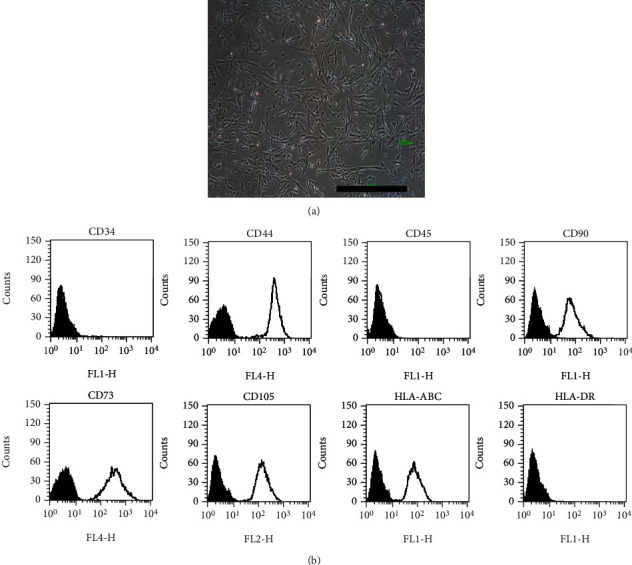
Morphology and surface markers of human umbilical cord mesenchymal stromal cells (HUCMSCs). (a) The morphology of HUCMSCs shows a fibroblastic-like appearance. (b) Surface marker panel of HUCMSCs. They are positive for HLA-ABC, CD44, CD90, CD73, and CD105 and negative for HLA-DR, CD34, and CD45.

The morphology and surface markers were used to validate the MSC characteristics of HUCMSCs [20]. The morphology of HUCMSC showed a fibroblast-like appearance (Figure 1(a)). The surface markers of HUCMSCs were positive for CD44, CD90, CD73, CD105, and HLA-ABC and negative for CD34, CD45, and HLA-DR (Figure 1(b)). Taken together, the HUCMSCs are in accordance with morphology and surface marker panels of MSCs.

#### 3.1.2. Trilineage Differentiation

**Figure 2:**
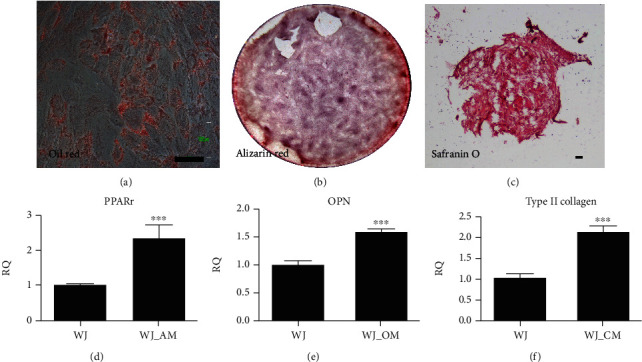
Three lineage differentiation of HUCMSCs. (a) Adipogenesis is for 2 weeks and is demonstrated by Oil red staining. Scale bar = 100 *μ*m. (b) Osteogenesis is for 2 weeks and is demonstrated by Alizarin red staining. (c) Chondrogenesis is for 3 weeks and is demonstrated by Safranin O staining. Scale bar = 100 *μ*m. (d–f) qPCR analysis of gene expression of three lineages: adipogenesis (*PPARr*), osteogenesis (*osteopontin* (*OPN*)), and chondrogenesis (*type II collagen*). ^∗∗∗^*p* < 0.001.

MSCs owned trilineage differentiation capability. After 14 days of adipogenic differentiation, the HUCMSC-differentiated adipocytes were positive with Oil red O staining, which revealed intracytoplasmic oil droplets (Figure 2(a)). After 14 days of osteogenic differentiation, the HUCMSC-differentiated osteoblasts showed positive staining for Alizarin red staining, which stained mineral deposits in cells (Figure 2(b)). After 21 days of chondrogenic differentiation, the HUCMSCs conglobated into a pellet (Figure 2(c)). The chondrogenic proteoglycan in the HUCMSC-differentiated chondrocytes was stained by Safranin O.

The gene expression assessed by qRT-PCR showed that *PPARγ* (an adipocyte gene, Figure 2(d)), *osteopontin* (an osteocyte gene, Figure 2(e)), and *type II collagen* (a chondrocyte gene, Figure 2(f)) increased expression after differentiation. The findings indicated that the HUCMSCs had a trilineage differentiation in vitro.

### 3.2. HA Enhances Chondrocyte-Related Gene Expression of HUCMSCs In Vitro

**Figure 3:**
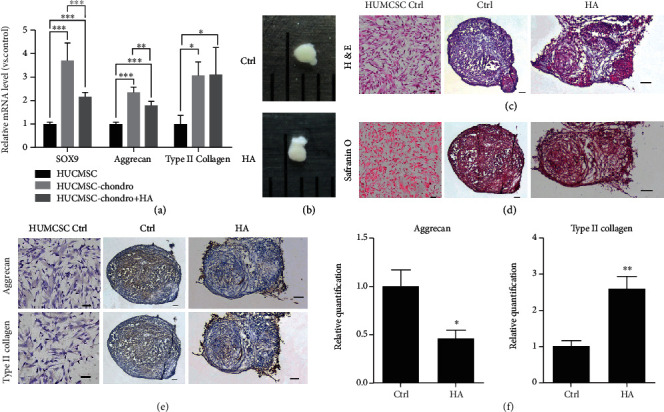
qRT-PCR and immunohistochemistry (IHC) of HUCMSCs after chondrogenic differentiation. (a) Expression of chondrogenic genes (*SOX9*, *aggrecan*, and *type II collagen*) in HUCMSCs is treated with or without hyaluronic acid (HA) compared to no differentiation HUCMSCs. (b) The gross picture of the pellet differentiated from HUCMSCs is treated with or without HA. (c) H&E staining of the pellet and HUCMSC (negative control). (d) Safranin O staining of the pellet and HUCMSC (negative control). (e) IHC of aggrecan and type II collagen of the pellet and HUCMSC (negative control). (f) The quantification of aggrecan and type II collagen. Scale bar = 100 *μ*m. ^∗^*p* < 0.05, ^∗∗^*p* < 0.01, and ^∗∗∗^*p* < 0.001. Ctrl: control.

The HUCMSCs were treated without or with (0% and 0.05%) HA and underwent chondrogenic differentiation in vitro for 3 weeks. The HA-treated HUCMSC-derived chondrocytes had significantly increased expression of chondrogenic markers, *SOX9*, *aggrecan*, and type II collagen (*COL2A1*) than undifferentiated HUCMSCs (Figure 3(a)). Figures 3(b)–3(d) show gross pictures, histology, and Safranin O staining of the pellet from HUCMSCs treated without and with HA and HUCMSCs. In immunohistochemistry (IHC) staining, HUCMSCs treated with HA exhibited significantly lower aggrecan (Figure 3(e)) and higher type II collagen (Figure 3(f)) expression than HUCMSCs without HA treatment. Taken together, HUCMSCs treated with HA could undergo chondrogenesis.

### 3.3. MRI of the Cartilage at 6 Weeks and 12 Weeks after Transplantation

A total of six 14-month-old NZW rabbits were used in this study. Both knees of each rabbit underwent ACLT procedure; the left knee acted as control and the right one was for HUCMSC therapy.

Eight weeks after ACLT, HUCMSC therapy was performed. A magnetic resonance imaging (MRI) study was performed for each knee in 6 weeks and 12 weeks after HUCMSC therapy.

**Figure 4:**
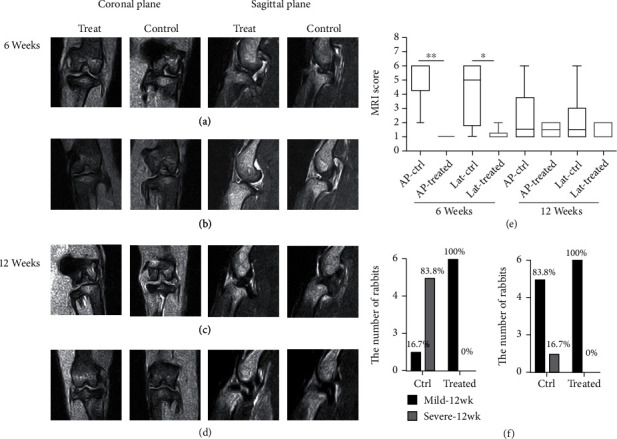
Knee joint MRI studies (HUCMSC 1 × 10^7^ cells+HA and normal saline control) at 6 weeks and 12 weeks (*n* = 6). (a) and (c) are taken from the same rabbit while (b) and (d) are from another. (e) The joints receiving 1 × 10^7^ HUCMSC+HA therapy show a significant decrease of the severity of osteoarthritis at 6 weeks than normal saline control knees, but they show no significant reduction of the severity of osteoarthritis at 12 weeks. AP: anterior-posterior view; Lat: lateral view; Ctrl: control. (f) The comparison of the number of rabbits and percentage of rabbits with mild and severe joint destruction diagnosed by MRI examination between normal saline control and treatment groups. Left panel and right panel revealed the lesion observed at 6 and 12 weeks after treatment, respectively. The *p* value in 6 and 12 weeks between the two groups is 0.008 and 0.5, respectively.

A 1 × 10^7^ HUCMSC plus 0.05% HA therapy was performed for each rabbit 8 weeks after ACLT. The knee joint MRI studies are shown in Figure 4 where (a) and (c) were taken from the same rabbit while (b) and (d) were from another. Figure 4(e) shows MRI grading scores. The joints that received 1 × 10^7^ HUCMSC+0.05% HA therapy showed reduced severity of osteoarthritis in 6 weeks (^∗^*p* < 0.05 in lateral view, ^∗∗^*p* < 0.001 in anteroposterior (AP) view). In 6 weeks, we found that 83.3% of the control joints showed severe osteoarthritis, and the other 16.7% joints showed mild osteoarthritis. Contrarily, 100% of the joints that received 1 × 10^7^ HUCMSC+HA therapy showed mild osteoarthritis (Figure 4(f)). The joints that received 1 × 10^7^ HUCMSC+HA therapy showed a significant decrease in the severity of osteoarthritis (*p* = 0.008) (Figure 4(f)). In 12 weeks, we found that 16.7% of the control joints showed severe osteoarthritis, and 83.3% joints showed mild osteoarthritis. Conversely, 100% of the joints that received 1 × 10^7^ HUCMSC+HA therapy showed mild osteoarthritis. The joints that received 1 × 10^7^ HUCMSC+HA therapy showed no significant reduction in the severity of osteoarthritis (*p* = 0.5) (Figure 4(f)). Taken together, HUCMSC and HA treatment could significantly recover cartilage defects under MRI examination after 6 weeks of transplantation.

### 3.4. Histology of Joint Cartilage

**Figure 5:**
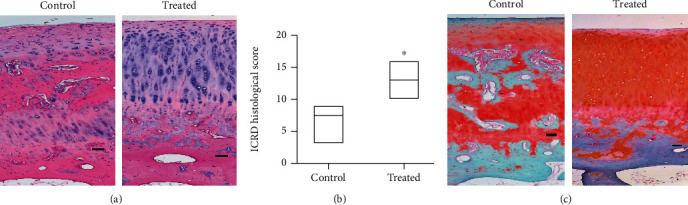
Histology and ICRS score of the rabbit osteoarthritis model after 12 weeks of experiments (*n* = 4). (a) Representative image of histology of the cartilage in normal saline control and HUCMSC+HA-treated knees. Scale bar = 100 *μ*m. (b) The normal saline control joints show a significantly lower ICRS histological score than the joints which receive 1 × 10^7^ HUCMSC+HA therapy (*n* = 4, *p* = 0.029). (c) The content of Safranin O staining after 12 weeks of experiments (*n* = 4). Representative image of the Safranin O staining in the cartilage of normal saline control and treated knee. Scale bar = 100 *μ*m. ^∗∗^*p* < 0.01.

The hematoxylin and eosin stain and Safranin O stain showed less cartilage destruction in the joints that received 1 × 10^7^ HUCMSC+0.05% HA therapy than in the control joints (Figures 5(a) and 5(c)). The joints that received 1 × 10^7^ HUCMSC+HA therapy showed significantly higher ICRS histological score (median, 13; range, 10-16) than the control joints (median, 3; range, 3-9) (*p* = 0.029) (Figure 5(b)).

### 3.5. Immunohistochemistry of Cartilage

**Figure 6:**
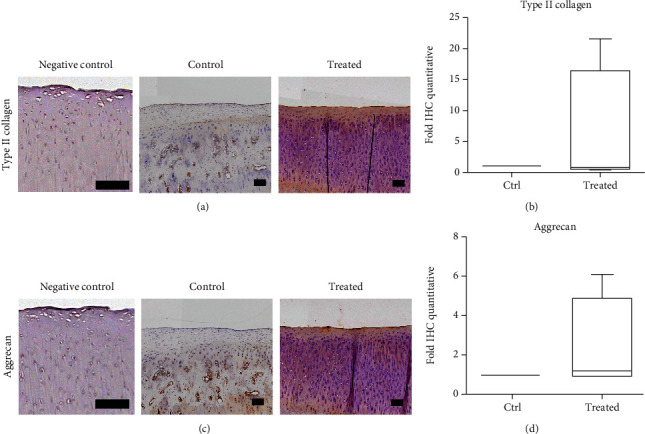
Immunohistochemistry of type II collagen and aggrecan of the cartilage after 12 weeks of experiments. (a) Representative image of type II collagen in the cartilage of normal cartilage (negative control), normal saline control, and HUCMSC+HA-treated knee. Scale bar = 100 *μ*m. (b) The 1 × 10^7^ HUCMSC+HA-treated joints show no significantly higher content of type II collagen than the normal saline control joints (*n* = 4). (c) Representative image of aggrecan in the cartilage of normal cartilage (negative control), normal saline control, and treated knee. Scale bar = 100 *μ*m. (d) The 1 × 10^7^ HUCMSC+HA-treated joints show no significantly higher content of aggrecan than the normal saline control joints (*n* = 4). No statistical differences are noted between both groups. Ctrl: control.

Nearly pale staining was noted in the normal cartilage (negative control) and normal saline control knee with type II collagen (Figure 6(a)) and aggrecan (Figure 6(c)), indicating absence of hyaline cartilage (Figures 6(a) and 6(c)). Conversely, the transplanted knee showed a more even distribution of staining which indicated hyaline cartilage presented (Figures 6(a) and 6(c), treated groups). However, the accumulated quantification of the staining intensity (*n* = 6) was not significant between the two groups (Figures 6(b) and 6(d)).

### 3.6. Inflammation of the Cartilage

**Figure 7:**
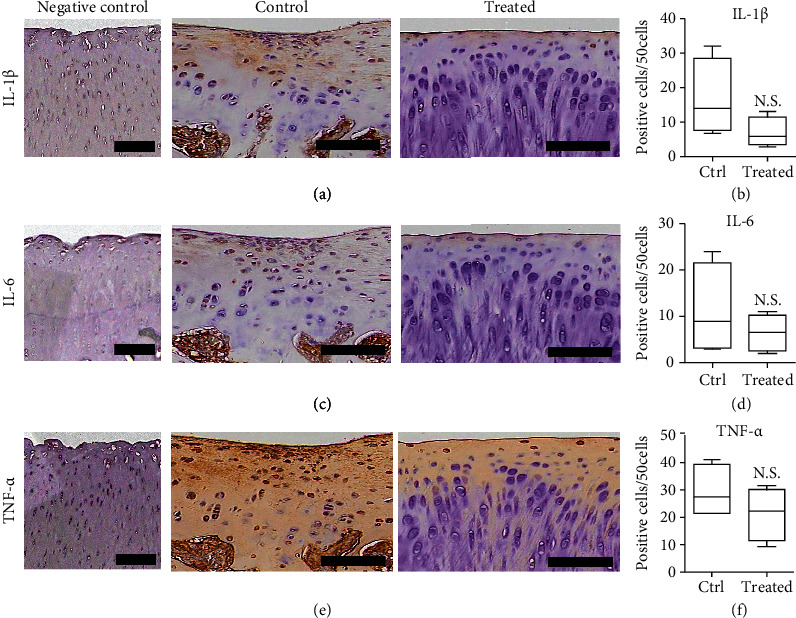
Immunohistochemistry of inflammatory markers TNF-*α*, IL1-*β*, and IL6 in the cartilage after 12 weeks of experiments. (a) Representative image of IL-1*β* in the cartilage of normal cartilage (negative control), normal saline control, and treated knee. Scale bar = 100 *μ*m. (b) The 1 × 10^7^ HUCMSC+HA-treated joints show no significantly higher content of IL-1*β* than the normal saline control joints (*n* = 4). (c) Representative image of IL-6 in the cartilage of normal cartilage (negative control), normal saline control, and treated knee. Scale bar = 100 *μ*m. (d) The 1 × 10^7^ HUCMSC+HA-treated joints show no significantly higher content of IL-6 than the normal saline control joints (*n* = 4). No differences are noted between both groups. (e) Representative image of TNF-*α* in the cartilage of normal cartilage (negative control), normal saline control, and treated knee. Scale bar = 100 *μ*m. (f) The 1 × 10^7^ HUCMSC+HA-treated joints show no significantly higher content of TNF-*α* than the normal saline control joints (*n* = 4). No statistical differences are noted between both groups. Ctrl: control.

We then checked immunostaining of inflammatory markers, including IL-1*β*, IL6, and TNF-*α* of the two groups (Figure 7). We also found decreasing expression of IL-1*β*, IL6, and TNF-*α* in the treated group. However, the quantification of the staining percentage of these inflammatory markers was not significant between the two groups (Figure 7).

### 3.7. MMP13 Decreased after HUCMSC Transplantation

**Figure 8:**
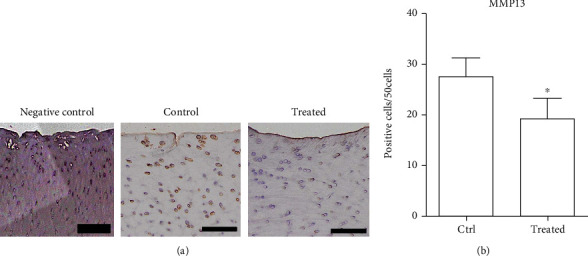
Immunohistochemistry of catabolic marker MMP13. (a) Representative image of MMP13 in the cartilage of normal cartilage (negative control), normal saline control, and HUCMSC+HA-treated knee. Scale bar = 100 *μ*m. (b) The 1 × 10^7^ HUCMSC+HA-treated joints show significantly lower expression of MMP13 than the normal saline control joints (*n* = 4). ^∗^*p* = 0.03 by the Student *t*-test. Ctrl: control.

We further checked immunostaining of a catabolic marker, MMP13, expression of the two groups (Figure 8). We found that MMP13 significantly decreased in the cartilage after HUCMSC+HA transplantation (*p* = 0.03). In addition, the decrease in cartilage destruction after HUCMSC+HA transplantation may be due to decreased MMP13 expression.

## 4. Discussion

In this study, we represented typical OA findings in this rabbit model. All the joints showed significant cartilage destruction after ACLT. We found that the HUCMSC combined with HA therapy showed significant improvement in the joints on histological findings. However, the MRI study could only detect the improvement in 6 weeks, but not in 12 weeks. There was no statistical difference between the two groups regarding aggrecan, type II collagen, and inflammatory markers. Lastly, we found that cartilage after HUCMSC transplantation showed a decrease in the expression of a catabolic marker (MMP13).

In preclinical models, intra-articular transplantation of MSCs showed a promising effect on resurfacing of degenerated cartilage surface [21]. Moreover, injection of MSCs intra-articularly decreased knee pain and protected cartilage noted in clinical trials [21]. Nevertheless, engraftment absent in the treated knee implied that there is another mechanism responsible for repairing the cartilage. The secretomes of MSCs include antiapoptosis, anti-inflammation, anticatabolic, and immunomodulation effects, which may be responsible for cartilage protection [5, 21]. Our study showed that histology of cartilage destruction was improved after transplantation of HUCMSCs and HA. However, IHC failed to demonstrate increasing type II collagen and aggrecan and decreasing inflammatory markers. Nevertheless, a catabolic marker, MMP13, was reduced after transplantation of HUCMSCs.

In synovial fluid, the quality and quantity of HA are changed and may be related to cartilage destruction during the OA process [22]. It is commonly used with HA as an intra-articular treatment for OA, although the results are variable. A systematic review revealed that HA is better than corticosteroids for OA treatment [23]. Conversely, the previous study found that there was no effect of HA compared to the placebo in treating cartilage destruction [24]. HA-only treatment might not be an ideal modality for OA. HA can promote cell migration and stem cell chondral differentiation [25]. In this study, we demonstrated that HUCMSCs and HA could induce more chondral differentiation.

SOX9, type II collagen, and aggrecan expressions are essential for chondrogenesis [26, 27]. The previous study showed that transfected SOX9 in bone marrow MSCs enhances chondrogenic differentiation [28]. Type II collagen is specific for cartilage tissue. The expression of type II collagen depends on SOX9, a major transcription factor for chondrogenesis [29]. Enhanced SOX9 expression also increases the expression of type II collagen and aggrecan [29]. In our study, the gene expressions of SOX9, type II collagen, and aggrecan increased after chondrogenic differentiation of HUCMSCs. However, after transplantation of HUCMSCs into the knee, the expressions of SOX9, aggrecan, and type II collagen were not significantly increased.

Inflammatory cells produced by cytokines may play an important role in cartilage destruction in OA [30]. A previous study showed that proinflammatory cytokines (TNF-*α*, IL-1*α*, and IL-6) could induce chondrocyte apoptosis [30]. IL-6 was found elevated in the synovial fluid of obese patients with OA [31]. Previous studies also showed that IL-6 inhibits type II collagen expression, which causes cartilage destruction [32]. IL-1*β* was reported to trigger chondrocyte apoptosis by increasing MMP3 and MMP13, which degrade the extracellular matrix [33]. IL-1*β*-inducing p38 MAPK signaling pathway was involved in the apoptosis of chondrocytes [34]. TNF-*α* is also involved in the apoptosis of chondrocytes [35]. TNF-*α* also can induce ADAMTS-4 via the p38 MAPK signaling pathway to increase cartilage degradation in OA [36]. Our study also investigated the expression of these proinflammatory cytokines in cartilage after transplantation of HUCMSCs. We showed that there was only a decreasing trend of these proinflammatory cytokines without statistical significance.

MMP13 is an enzyme responsible for cartilage degradation. MMP13 can target type II collagen, type IV and type IX collagen, perlecan, and osteonectin in cartilage for degradation [15]. Patients with OA also presented with high expression of MMP13 in destructed cartilage [37]. MMP13-overexpression transgenic mice also spontaneously developed an OA cartilage destruction phenotype [38]. Another study used MMP13 transgenic mice to study the effect of MMP13-inhibiting agents on OA progression. They found that using an MMP13-inhibiting agent could effectively decelerate cartilage destruction in a mouse OA model [39]. Our study also showed via IHC of MMP13 in cartilage decreasing after HUCMSC+HA treatment.

MRI can measure the microscopic components of cartilage, especially in the early stage of OA, with noninvasive, quantitative, and objective ways [40, 41]. The measurements include longitudinal and transverse relaxation times (T1 and T2 images). T1 image is reported as a biomarker for water and macromolecular content [42]. T2 image is linked to the hydration and collagen fiber content in articular cartilage [43, 44]. MRI can detect the cartilage in the early phase of its destruction in the rabbit OA model (can detect early at 4 weeks post-ACLT surgery) [45]. Recently, MRI was reported to detect cartilage lesions early at two weeks after surgery [46]. In our study, we only detected cartilage destruction at 6 weeks and 12 weeks after surgery. We found a significant reduction of lesions in the treated group at 6 weeks but not at 12 weeks.

There were several limitations in this study. First, the number of rabbits was limited, and more rabbits may be needed to confirm the results. Second, the resolution of MRI was low in our study. A high resolution, such as 9 T, would be better for the early detection of early cartilage defects [46]. Third, the IHC findings showed no significant results, which may be due to the small sample size.

In conclusion, transplantation of HUCMSCs and HA could attenuate cartilage destruction in osteoarthritis. Our study provides evidence for future clinical trials.

## Data Availability

All data were in the manuscript.

## References

[1] Hunter D. J., Bierma-Zeinstra S. (2019). Osteoarthritis. *Lancet*.

[2] Wallace I. J., Worthington S., Felson D. T. (2017). Knee osteoarthritis has doubled in prevalence since the mid-20th century. *Proceedings of the National Academy of Sciences of the United States of America*.

[3] Latourte A., Kloppenburg M., Richette P. (2020). Emerging pharmaceutical therapies for osteoarthritis. *Nature Reviews Rheumatology*.

[4] Bannuru R. R., Osani M. C., Vaysbrot E. E. (2019). OARSI guidelines for the non-surgical management of knee, hip, and polyarticular osteoarthritis. *Osteoarthritis and Cartilage*.

[5] Chang Y.-H., Liu H.-W., Wu K.-C., Ding D.-C. (2016). Mesenchymal stem cells and their clinical applications in osteoarthritis. *Cell Transplantation*.

[6] Harrell C. R., Markovic B. S., Fellabaum C., Arsenijevic A., Volarevic V. (2019). Mesenchymal stem cell-based therapy of osteoarthritis: current knowledge and future perspectives. *Biomedicine & Pharmacotherapy*.

[7] Kim S. H., Ha C. W., Park Y. B., Nam E., Lee J. E., Lee H. J. (2019). Intra-articular injection of mesenchymal stem cells for clinical outcomes and cartilage repair in osteoarthritis of the knee: a meta-analysis of randomized controlled trials. *Archives of Orthopaedic and Trauma Surgery*.

[8] Ding D.-C., Chang Y.-H., Shyu W.-C., Lin S.-Z. (2015). Human umbilical cord mesenchymal stem cells: a new era for stem cell therapy. *Cell Transplantation*.

[9] Yan H., Yu C. (2007). Repair of full-thickness cartilage defects with cells of different origin in a rabbit model. *Arthroscopy*.

[10] Ha C. W., Park Y. B., Chung J. Y., Park Y. G. (2015). Cartilage repair using composites of human umbilical cord blood-derived mesenchymal stem cells and hyaluronic acid hydrogel in a minipig model. *Stem Cells Translational Medicine*.

[11] Lee K. B. L., Hui J. H. P., Song I. C., Ardany L., Lee E. H. (2007). Injectable mesenchymal stem cell therapy for large cartilage defects--a porcine model. *Stem Cells*.

[12] Gupta R. C., Lall R., Srivastava A., Sinha A. (2019). Hyaluronic acid: molecular mechanisms and therapeutic trajectory. *Front Vet Sci*.

[13] Wu K.-C., Chang Y.-H., Liu H.-W., Ding D.-C. (2018). Transplanting human umbilical cord mesenchymal stem cells and hyaluronate hydrogel repairs cartilage of osteoarthritis in the minipig model. *Tzu Chi Medical Journal*.

[14] Ding D.-C., Wu K. C., Chou H. L., Hung W. T., Liu H. W., Chu T. Y. (2015). Human infrapatellar fat pad-derived stromal cells have more potent differentiation capacity than other mesenchymal cells and can be enhanced by hyaluronan. *Cell Transplantation*.

[15] Shiomi T., Lemaître V., D’Armiento J., Okada Y. (2010). Matrix metalloproteinases, a disintegrin and metalloproteinases, and a disintegrin and metalloproteinases with thrombospondin motifs in non- neoplastic diseases. *Pathology International*.

[16] Livak K. J., Schmittgen T. D. (2001). Analysis of relative gene expression data using real-time quantitative PCR and the 2^−*ΔΔ* _C_^_T_ method. *Methods*.

[17] Batiste D. L., Kirkley A., Laverty S., Thain L. M. F., Spouge A. R., Holdsworth D. W. (2004). _Ex vivo_ characterization of articular cartilage and bone lesions in a rabbit ACL transection model of osteoarthritis using MRI and micro-CT. *Osteoarthritis and Cartilage*.

[18] Mainil-Varlet P., Aigner T., Brittberg M. (2003). Histological assessment of cartilage repair: a report by the Histology Endpoint Committee of the International Cartilage Repair Society (ICRS). *The Journal of Bone and Joint Surgery. American Volume*.

[19] Ichiseki T., Shimasaki M., Ueda Y. (2018). Intraarticularly-injected mesenchymal stem cells stimulate anti-inflammatory molecules and inhibit pain related protein and chondrolytic enzymes in a monoiodoacetate-induced rat arthritis model. *International Journal of Molecular Sciences*.

[20] Dominici M., le Blanc K., Mueller I. (2006). Minimal criteria for defining multipotent mesenchymal stromal cells. The International Society for Cellular Therapy position statement. *Cytotherapy*.

[21] Mancuso P., Raman S., Glynn A., Barry F., Murphy J. M. (2019). Mesenchymal stem cell therapy for osteoarthritis: the critical role of the cell secretome. *Frontiers in Bioengineering and Biotechnology*.

[22] Venable R. O., Stoker A. M., Cook C. R., Cockrell M. K., Cook J. L. (2008). Examination of synovial fluid hyaluronan quantity and quality in stifle joints of dogs with osteoarthritis. *American Journal of Veterinary Research*.

[23] Iannitti T., Lodi D., Palmieri B. (2011). Intra-articular injections for the treatment of osteoarthritis: focus on the clinical use of hyaluronic acid. *Drugs in R & D*.

[24] Altman R. D., Akermark C., Beaulieu A. D., Schnitzer T. (2004). Efficacy and safety of a single intra-articular injection of non-animal stabilized hyaluronic acid (NASHA) in patients with osteoarthritis of the knee. *Osteoarthritis and Cartilage*.

[25] Li L., Duan X., Fan Z. (2018). Mesenchymal stem cells in combination with hyaluronic acid for articular cartilage defects. *Scientific Reports*.

[26] Hardingham T. E., Oldershaw R. A., Tew S. R. (2006). Cartilage, SOX9 and Notch signals in chondrogenesis. *Journal of Anatomy*.

[27] Yi S. W., Kim H. J., Oh H. J. (2018). Gene expression profiling of chondrogenic differentiation by dexamethasone-conjugated polyethyleneimine with SOX trio genes in stem cells. *Stem Cell Research & Therapy*.

[28] Venkatesan J. K., Meng W., Rey-Rico A. (2020). Enhanced chondrogenic differentiation activities in human bone marrow aspirates via sox9 overexpression mediated by pNaSS-grafted PCL film-guided rAAV gene transfer. *Pharmaceutics*.

[29] Yasuda H., Oh C., Chen D., de Crombrugghe B., Kim J.-H. (2017). A novel regulatory mechanism of type II collagen expression via a SOX9-dependent enhancer in intron 6∗. *The Journal of Biological Chemistry*.

[30] Schuerwegh A. J., Dombrecht E. J., Stevens W. J., van Offel J. F., Bridts C. H., de Clerck L. S. (2003). Influence of pro-inflammatory (IL-1*α*, IL-6, TNF-*α*, IFN-*γ*) and anti- inflammatory (IL-4) cytokines on chondrocyte function. *Osteoarthritis and Cartilage*.

[31] Pearson M. J., Herndler-Brandstetter D., Tariq M. A. (2017). IL-6 secretion in osteoarthritis patients is mediated by chondrocyte- synovial fibroblast cross-talk and is enhanced by obesity. *Scientific Reports*.

[32] Porée B., Kypriotou M., Chadjichristos C. (2008). Interleukin-6 (IL-6) and/or soluble IL-6 receptor down-regulation of human type II collagen gene expression in articular chondrocytes requires a decrease of Sp1∗Sp3 ratio and of the binding activity of both factors to the _COL2A1_ promoter∗. *The Journal of Biological Chemistry*.

[33] Aida Y., Maeno M., Suzuki N., Shiratsuchi H., Motohashi M., Matsumura H. (2005). The effect of IL-1*β* on the expression of matrix metalloproteinases and tissue inhibitors of matrix metalloproteinases in human chondrocytes. *Life Sciences*.

[34] Yang G., Fan L., Tian S. J., Ding S., Luo J., Zheng J. (2017). Polydatin reduces IL-1*β*-induced chondrocytes apoptosis and inflammatory response via p38 MAPK signaling pathway in a rat model of osteoarthritis. *International Journal of Clinical and Experimental Medicine*.

[35] Aizawa T., Kon T., Einhorn T. A., Gerstenfeld L. C. (2001). Induction of apoptosis in chondrocytes by tumor necrosis factor-alpha. *Journal of Orthopaedic Research*.

[36] Xue J., Wang J., Liu Q., Luo A. (2013). Tumor necrosis factor-*α* induces ADAMTS-4 expression in human osteoarthritis chondrocytes. *Molecular Medicine Reports*.

[37] Roach H. I., Yamada N., Cheung K. S. C. (2005). Association between the abnormal expression of matrix-degrading enzymes by human osteoarthritic chondrocytes and demethylation of specific CpG sites in the promoter regions. *Arthritis and Rheumatism*.

[38] Neuhold L. A., Killar L., Zhao W. (2001). Postnatal expression in hyaline cartilage of constitutively active human collagenase-3 (MMP-13) induces osteoarthritis in mice. *The Journal of Clinical Investigation*.

[39] Wang M., Sampson E. R., Jin H. (2013). MMP13 is a critical target gene during the progression of osteoarthritis. *Arthritis Research & Therapy*.

[40] Guermazi A., Alizai H., Crema M. D., Trattnig S., Regatte R. R., Roemer F. W. (2015). Compositional MRI techniques for evaluation of cartilage degeneration in osteoarthritis. *Osteoarthritis and Cartilage*.

[41] Thüring J., Linka K., Itskov M. (2018). Multiparametric MRI and computational modelling in the assessment of human articular cartilage properties: a comprehensive approach. *BioMed Research International*.

[42] Berberat J. E., Nissi M. J., Jurvelin J. S., Nieminen M. T. (2009). Assessment of interstitial water content of articular cartilage with _T_ _1_ relaxation. *Magnetic Resonance Imaging*.

[43] Nissi M. J., Rieppo J., Töyräs J. (2006). _T_ _2_ relaxation time mapping reveals age- and species- related diversity of collagen network architecture in articular cartilage. *Osteoarthritis and Cartilage*.

[44] Nieminen M. T., Rieppo J., Töyräs J. (2001). T2 relaxation reveals spatial collagen architecture in articular cartilage: a comparative quantitative MRI and polarized light microscopic study. *Magnetic Resonance in Medicine*.

[45] Rautiainen J., Nissi M. J., Liimatainen T., Herzog W., Korhonen R. K., Nieminen M. T. (2014). Adiabatic rotating frame relaxation of MRI reveals early cartilage degeneration in a rabbit model of anterior cruciate ligament transection. *Osteoarthritis and Cartilage*.

[46] Kajabi A. W., Casula V., Ojanen S. (2020). Multiparametric MR imaging reveals early cartilage degeneration at 2 and 8 weeks after ACL transection in a rabbit model. *Journal of Orthopaedic Research*.

